# Tele-Rehabilitation Program in Idiopathic Pulmonary Fibrosis—A Single-Center Randomized Trial

**DOI:** 10.3390/ijerph181910016

**Published:** 2021-09-23

**Authors:** Jose Cerdán-de-las-Heras, Fernanda Balbino, Anders Løkke, Daniel Catalán-Matamoros, Ole Hilberg, Elisabeth Bendstrup

**Affiliations:** 1 Center for Rare Lung Diseases, Department of Respiratory Diseases and Allergy, Aarhus University Hospital, 8200 Aarhus, Denmark; karbends@rm.dk; 2 Department of Research and Development, Physio R&D ApS, 2000 Frederiksberg, Denmark; fernanda@physiord.com; 3 Department of Respiratory Medicine, Vejle Hospital, 7100 Vejle, Denmark; aloekke@gmail.com (A.L.); ole.hilberg@rsyd.dk (O.H.); 4 UC3M Medialab, Department of Communication and Media Studies, Madrid University Carlos III, 28903 Madrid, Spain; dacatala@hum.uc3m.es; 5 Health Research Centre, University of Almeria, 04120 Almeria, Spain

**Keywords:** tele-rehabilitation, idiopathic pulmonary fibrosis, virtual reality, virtual agent

## Abstract

We investigated the usefulness and effectiveness of tele-rehabilitation on exercise capacity in patients with idiopathic pulmonary fibrosis (IPF). A randomized study was carried out, including stable patients with IPF for 3 months of tele-rehabilitation: video and chat consultations with a physiotherapist and workout sessions with a virtual physiotherapist agent (VAPA). Moreover, 6-min walk test distance (6MWTD), forced vital capacity (FVC), diffusion capacity for carbon monoxide (DLCO), 7 days pedometry, Saint George Respiratory Questionnaire for interstitial lung disease, The King’s Brief Interstitial Lung Disease Questionnaire, and General Anxiety Disorder 7 Questionnaire were tested before and after 3 months of tele-rehabilitation, as well as after 3 and 6 months follow-up. Patient satisfaction and adherence were also measured for tele-rehabilitation with VAPA. Twenty-nine patients aged 70.9 ± 8.6 years, male 72.4%, FVC% 83.5 ± 17.7, DLCO% 50.6 ± 13.0, 6MWTD 468.4 ± 14.8 were included. Fifteen patients were randomized to tele-rehabilitation with VAPA and 14 to the control group. Differences in 6MWTD between groups were at baseline (+10 m (*p* = 0.11)) and after 3 (+39.5 m (*p* = 0.03)), 6 (+34.3 m (*p* = 0.02)), and 9 months (+40.5 m (*p* = 0.15)) follow-up. No difference was observed in pedometry and quality of life. Adherence was above 63%. Patient satisfaction was high. Tele-rehabilitation with VAPA appears to be useful in patients with IPF. Exercise capacity was better at follow up at 3 and 6 months compared with the control group. There was no change in quality of life or pedometry. Adherence and patient satisfaction were all high.

## 1. Introduction

Idiopathic pulmonary fibrosis (IPF) is a rare progressive fibrotic lung disease with a poor prognosis [1]. The adjusted incidence and prevalence of IPF is in the range of 0.09–1.30 and 0.33–4.51 per 10,000 persons, respectively [2]. The prognosis is poor with a median survival of 2–4 years without antifibrotic treatment and the majority of individuals die from respiratory causes, either infections or IPF progression [1]. There is no cure for the disease, but antifibrotic treatment slows down disease progression [1]. With time, increased fibrosis leads to worsening of gas exchange, resulting in hypoxemia during physical activity. This can cause dyspnea; fatigue; and reduction in physical activity, exercise tolerance, muscle strength, and quality of life (QoL) [3], which may lead to immobility, dependency on help from others, and social isolation [1,4].

In patients with IPF, pulmonary rehabilitation (PR) can improve dyspnea, quality of life, and functional exercise capacity in both the short and long term [5]. Rehabilitation programs for patients with IPF lasting 8–12 weeks are mostly offered in hospitals [4,6]. However, not all patients with IPF are suitable candidates for or wish to participate in these programs. Among reasons for non-participation are shielding owing to risk of infection, long travelling distance to rehabilitation facilities and severely compromised mobility [1]. Therefore, tele-rehabilitation may offer an alternative for patients with IPF to ensure participation in rehabilitation programs.

Tele-rehabilitation is flexible, allowing patients to exercise whenever they find it suitable, and it can reach patients across long distances as well as increase disease awareness [7]. In other diseases such as chronic heart disease and asthma, tele-rehabilitation has been at least as effective as standard rehabilitation and sustained long-term benefits have been demonstrated [7]. Different technologies and tele-rehabilitation platforms are available. Most are based on videoconferencing with one or more patients training together in real time with a therapist or based on virtual reality technologies with agents showing the exercises [8,9,10,11,12].

Our aim was to assess adherence, usefulness, and patient satisfaction of tele-rehabilitation, and to compare the effect of tele-rehabilitation to usual care without rehabilitation in patients with IPF. A new platform, the virtual autonomous physiotherapist agent (VAPA), was used to deliver the tele-rehabilitation.

## 2. Materials and Methods

### 2.1. Study Design

The study was a single-center, prospective, randomized clinical trial. Randomization was undertaken electronically at randomization.com (www.randomization.com, (accessed on 20 September 2021)). All subjects were randomized into one block (reproducible using seed 26058).

### 2.2. Study Participants

Stable patients with IPF were included between September 2017 and April 2018 from the outpatient clinic at Center for Rare Lung Diseases, Department of Respiratory Diseases and Allergy, Aarhus University Hospital, Denmark. Patients were eligible for inclusion if they had a diagnosis of IPF according to ATS/ERS/JRS/ALAT guidelines based on high-resolution computer tomography (HRCT) scan, lung function tests, bronchoscopy, and lung biopsies, if available [13]. Inclusion criteria were as follows: diffusion capacity for carbon monoxide (DLCO) ≥ 30% predicted, forced vital capacity (FVC) ≥ 50% predicted, six-minute walk test distance (6MWTD) ≥ 150 m, clinically stable (absolute decline in DLCO and FVC of less than 10% in the past 6 months), at least 18 years of age, and provided written informed consent. Patients were excluded if they had participated in a rehabilitation program in the previous six months, had musculoskeletal disorders or severe cardiac disease (ejection fraction < 30%, daily angina, and/or symptomatic valve disorders) that could compromise training, if they had conditions that could hamper the use of tele-rehabilitation, were unable to speak and/or understand Danish, and if they displayed unwillingness to implement the protocol.

Patients were randomized 1:1 to a 12-week tele-rehabilitation program on top of usual care or to a control group receiving only usual care (without rehabilitation). After 12 weeks of rehabilitation, patients in the intervention arm were offered daily use of VAPA during follow-up, but without physiotherapist guidance. End parameters were measured at baseline, after the end of the three-month tele-rehabilitation period (3 months from baseline), and again after three and six months of follow-up (6- and 9 months from baseline respectively).

### 2.3. Tele-Rehabilitation

**Figure 1:**
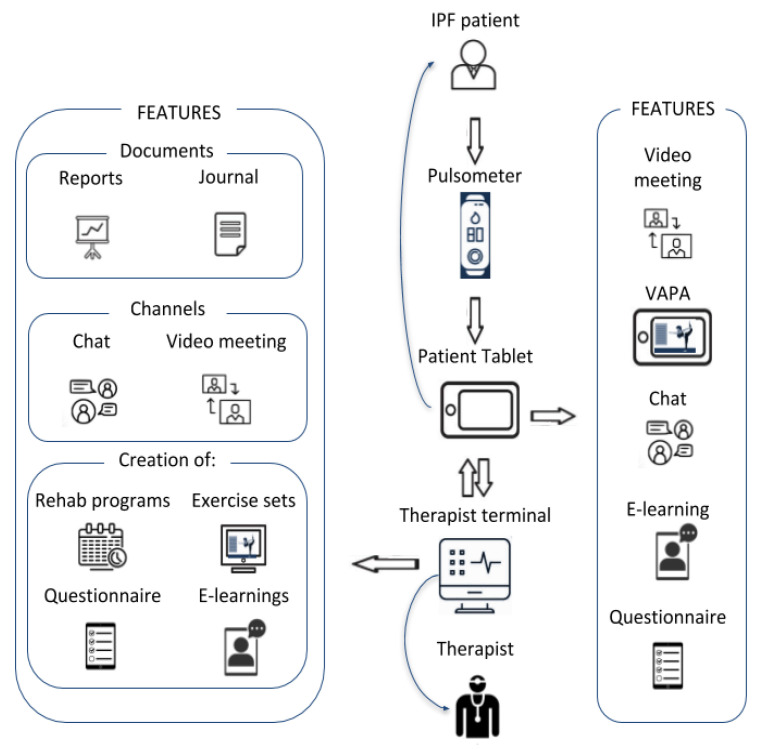
Digital ecosystem of the multidimensional VAPA platform.

Tele-rehabilitation was delivered using a virtual autonomous physiotherapist agent (VAPA), a platform developed in a European consortium (Physio R&D and Cortrium, Denmark and bookBeo and Laster Technologies, France) and two university hospitals (Aarhus University Hospital, Denmark and Oulu University Hospital, Finland) and funded by the Eurostars program [14]. The tele-rehabilitation program was originally developed based on feedback from patients with chronic cardiopulmonary diseases [15,16]. VAPA consists of (a) multidimensional software that serves as a service platform for therapists to create customized tele-rehabilitation programs for patients and with the ability to unify video consultations, e-learning packages, physical exercise programs, online questionnaires, patient digital files, and real-time chat function in the same tool [17] and (b) a mobile app for patients to install on a smartphone or tablet connected directly to a biometric sensor attachable to the patient’s chest, arms, or fingers to collect physical data like oximetry and heart rate, enabling the rehabilitation program to adjust in real time (Figure 1) [18]. Study participants received a smart tablet for the study and were instructed in its use.

The content of the tele-rehabilitation program is shown in Table 1.

### 2.4. Endpoints

The primary endpoint was exercise capacity measured as 6MWTD in meters (m) from baseline to 3 months (end of tele-rehabilitation) [26]. Secondary endpoints were 6MWTD from baseline to 6 months (mid follow up) and from baseline to 9 months (end follow up), pedometry measured as number of steps walked and total vector magnitude counts per minute score tracked by ActiGraph Monitor wGT3X-BT) for 7 days, [27] quality of life measured by the IPF-specific version of St. George’s Respiratory Questionnaire (SGRQ-I) [28], King’s Brief Interstitial Lung disease (K-BILD) in % [29], and the General Anxiety Disorder Score (GAD7) in a score from 1–7 [30]. Pulmonary function was measured as forced vital capacity (FVC) in % and diffusion capacity for carbon monoxide (DLCO) in %. Patients in the tele-rehabilitation with VAPA group were asked to rate their satisfaction with VAPA by answering a five-point likert scale after each training session, where 1 is the worst score. The training time per exercise set executed and the weekly average training time were registered. This was compared to the minimal training time per week reported to have a satisfactory value for IPF patients, and 60 min weekly training was targeted as the minimum training time [3].

### 2.5. Statistics

There are no available studies of tele-rehabilitation in IPF, and previous studies used other QoL measures. Thus, there are no reliable results on which to base a sample size calculation. Our study was planned as a pilot study to estimate the effect of a tele-rehabilitation solution, including 24 patients divided into two groups with 12 in each group. A drop-out rate of 25% was expected, thus we aimed to recruit 30 patients in total.

Changes in endpoints after tele-rehabilitation and follow-up were compared between groups using the unpaired t-test for parametric data and Mann–Whitney rank-sum test non-parametric data using 95% confidence intervals. Changes from baseline to after both tele-rehabilitation and follow-up were analyzed within groups using the paired t-test for parametric data and the Wilcoxon signed-rank test for non-parametric data. Data results are expressed as means ± standard deviation The analysis was run in the R program and IBM SPSS Statistics version 25. All results were analyzed using the intention-to-treat approach [31].

## 3. Results

### 3.1. Patients

**Figure 2:**
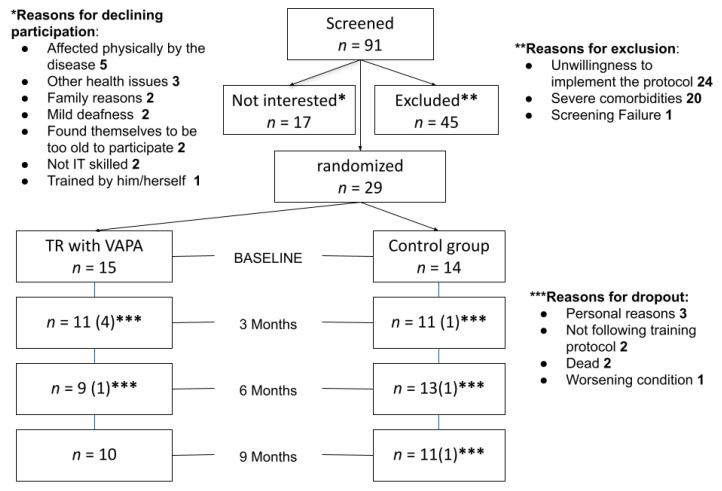
Enrollment and randomization in the overall population.

Ninety-one patients with IPF were screened for participation and twenty-nine patients were included. Fifteen patients were randomized to tele-rehabilitation with VAPA and 14 patients to the control group (Figure 2). Mean age was 70.1 ± 8.8 and 72.4 ± 7.6 in the tele-rehabilitation with VAPA and control groups, respectively. Baseline characteristics revealed numerically, although not statistically significantly more males, a longer disease duration before inclusion, and a higher number of ever-smokers in the tele-rehabilitation with VAPA group; FVC% predicted was by chance lower in the tele-rehabilitation with VAPA group (mean difference −14.1%, *p*-value 0.03); all other baseline parameters were not statistically significant different between groups. Baseline demographics are displayed in Table 2.

Eligible patients declining participation suffered from more advanced lung disease (Table S1 in supplementary File S1). Excluded patients unwilling to implement the protocol wanted to participate only in the intervention arm.

### 3.2. Endpoints

**Figure 3:**
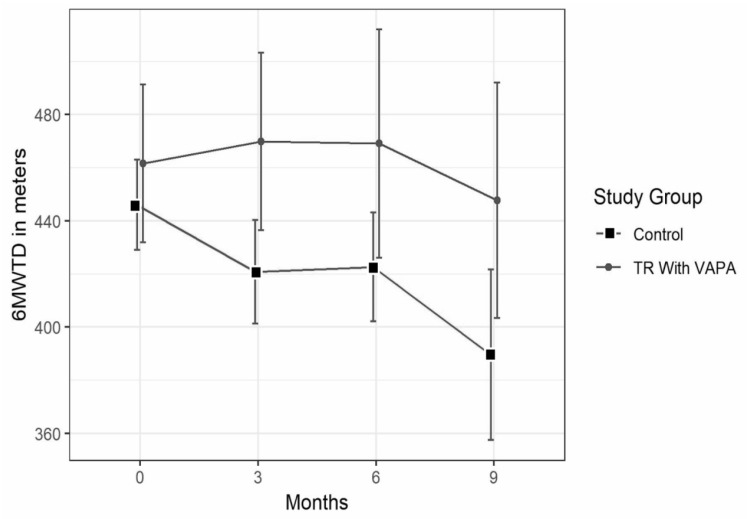
Six-minute walk test distance (6MWTD) in the tele-rehabilitation with VAPA group and the control group. Data are displayed as the mean difference in meters and standard deviations.

Tele-rehabilitation with VAPA resulted in sustained 6MWTD of 465.64 ± 122.75 at baseline compared with 469.91 ± 115.63 after 3 months, 469.10 ± 135.88 at 6 months, and 447.67 ± 132.97 at 9 month, while controls experienced a decline from baseline at 456.00 ± 51.73 to 420.80 ± 70.16 after 3 months, 422.6 ± 76 after 6 months, and 389.6 ± 84.8 at 9 months (Figure 3). Statistically significant differences between groups were found at 3 months—(+39.5 m, *p* = 0.03) and at 6 months from baseline (+34.3 m, *p* = 0.02), but not at 9 months from baseline (+40.0 m, *p* = 0.15).

No statistically significant differences between groups were observed regarding exercise activity as measured by pedometry, QoL, and pulmonary function test (PFT) (Table 3). No adverse events were reported.

### 3.3. Training after End of Rele-Rehabilitation

No participants in the control group reported any form of training during the study. In the tele-rehabilitation with VAPA group, five and three patients reported continuous training with the tele-rehabilitation with VAPA program, but without interaction with a therapist after 6 and 9 months, respectively.

### 3.4. Adherence and Patient Satisfaction

**Figure 4:**
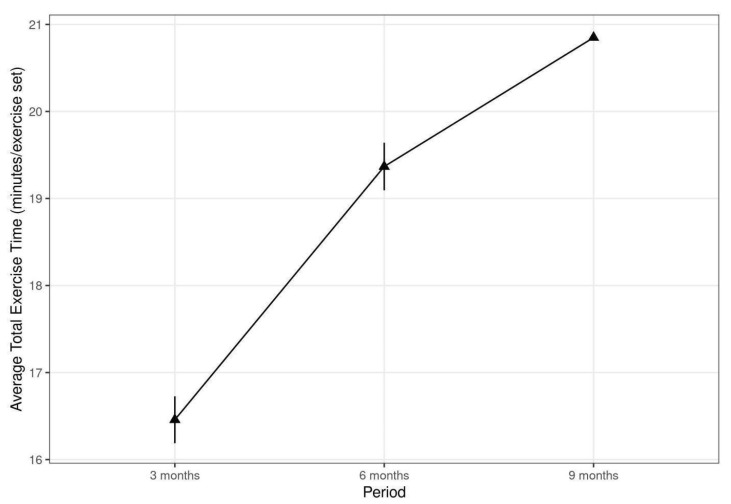
Average time per exercise session in the tele-rehabilitation with VAPA group for each 3-month period.

As shown in Table 4 and Figure 4, adherence to exercise for the patients in the tele-rehabilitation with VAPA group increased over time. During the first three months, 15 patients had an adherence of 64% with an average of 16.5 min of exercise per session. Between 3 and 6 months, 5 patients decided to continue training on their own with an adherence of 108%, and trained on average 19.5 min per session. Three patients decided to continue training between 6 and 9 months, with an adherence of 110%, and trained 21 min per session. A total of 168 responses on the Likert satisfaction score (1–5) resulted in an average score of 3.8 ± 0.5.

Additional results from this study regarding total group baseline data, results on all variables in relation to pulmonary function, physical performance and quality of life in control and Tele-rehabilitation with VAPA groups and comparison between groups can be found in the Supplementary File S1.

## 4. Discussion

Our aim was to assess adherence, usefulness, and patient satisfaction of tele-rehabilitation, as well as to compare the effect of tele-rehabilitation to usual care without rehabilitation in patients with IPF. This new platform, the virtual autonomous physiotherapist agent (VAPA), appears to be useful. We found significant differences between groups after 3- and 6 months from baseline. No difference was observed in pedometry and quality of life. In addition, the study shows high adherence and patient satisfaction. This study is the first to evaluate the usefulness of a new platform, VAPA, for tele-rehabilitation in patients with IPF as an alternative to standard care. Tele-rehabilitation with VAPA sustained exercise capacity and showed high patient adherence and satisfaction.

According to a recent review, rehabilitation in patients with IPF resulted in improved exercise capacity, with a mean difference of 6MWTD of 40.07 m compared with controls [5]. Our results showed a mean difference of 39.5 m, suggesting that tele-rehabilitation with VAPA might have a potential for improving exercise capacity in patients with IPF [3,32]. The improvement is similar to the minimal clinically important difference in patients with IPF [33].

In our study, training with tele-rehabilitation seemed to somewhat stabilize physical capacity, while the control group continuously deteriorated in 6MWTD as time advanced [34]. Pulmonary function tests remained unchanged, supporting that patients in both groups were stable with respect to IPF severity during the study. Previous studies with 8 or 12 weeks of rehabilitation with 60 min of weekly training have demonstrated improved exercise capacity [3,32]. Contrary to previous studies, exercise capacity measured as the 6MWTD was maintained after the end of rehabilitation in our study. This result should be interpreted with caution owing to the low number of patients but could suggest that tele-rehabilitation with VAPA may have the potential to support continued training.

Hospital-based pulmonary rehabilitation programs combine aerobic exercise, respiratory muscle training, oxygen therapy, nutritional intervention, education, and self-management approaches, among others. In tele-rehabilitation with VAPA, we digitalized the content of the rehabilitation program as much as possible, but still included aerobic exercise and respiratory muscle training with nutritional intervention, education, and self-management approaches by creating e-learning packages with direct messages and “know how” on how to cope with disease symptoms. We were not able to show improved QoL, in accordance with most other studies [3,32,35], but contrary to Yu et al. [36], who found improved QoL and FVC% in a review regarding pulmonary rehabilitation. Again, this might be caused by the pilot study design, and larger prospective studies are needed. Future studies should compare tele-rehabilitation with VAPA to standard rehabilitation and explore if virtual programs such as tele-rehabilitation with VAPA can be upgraded with (a) interaction with other biometric variables such as oximetry, blood pressure, and breath rate to customize the exercise training even further and (b) more qualified e-learning packages addressing specific needs of patients with IPF to increase behavioral changes and QoL.

Regarding the 7 days pedometry and 7 dVMCPM, we were not able to detect any changes between groups, but we observed a trend, though statistically insignificant, towards better performance in the tele-rehabilitation with VAPA group. The low number of patients and the fact that patients started the study in different seasons of the year may partly explain this. For instance, a patient enrolled during the summer may potentially have walked less at follow-up in the winter.

Considering the different tele-rehabilitation platforms that are available [8,9,10,11,12], our study showed that the agent–avatar interaction following the biometric data of the patients used in our study is another technology that seems promising.

In virtual reality, the avatar is a representation of the patient in the virtual world. The avatar interacts with the virtual agent, a computer-based entity who guides the user to perform the intended tasks. An avatar can, for instance, be a graphical representation of a patient’s body shape, or a sound representing the pulse of the patient, whereas an agent can be presented by a human-like graphical representation or just a voice. Importantly, the avatar, as a representation of the patient, is followed continuously by the agent, giving the agent the opportunity to know where the patient is in real time when performing a certain task. Such a capability triggers behavioral changes in the agent who follows specific rules programmed in a decision support system. Rules are based on avatars’ behavioral changes.

In the VAPA set-up aimed at patients with cardio-pulmonary diseases, the best parameter to follow is the heart rate. Here, the heart rate creates a numerical avatar in the VAPA agent virtual world and triggers the agent’s guidance according to whether the patient’s heart rate is above or below a certain pre-specified threshold during exercise performance.

Video platforms such as the one used in the studies by Hansen and Rayce [37,38,39] allow patients to stay at home and meet virtually. The aim of such an approach is to enable patients to participate online in the pulmonary rehabilitation at the hospital and to let the telemediated training support the social needs of patients, like meeting face-to-face at the hospital. However, the social benefits experienced in conventional hospital group training programs could not be transferred to a video training set-up [40]. Whether the lack of socializing with other patients influences QoL needs to be examined. Tele-rehabilitation is convenient as it allows patients to train at home, but video training is often scheduled at fixed time points just as in the hospital setting, and thus is less flexible compared with the VAPA platform. Patients still have to plan their day and energy according to the training appointment [39], contrary to VAPA, where patients can train when they like.

Having to attend training at scheduled appointments virtually at home can also affect patients’ partners who need to change their way of living as they may need to be available in the case of problems with the ongoing video meeting [39]. Moreover, physiotherapists report difficulties with observing the skin color of the patients, i.e., cyanosis or pallor, during video conferences. As a result, online exercise intensity is lower compared with hospital training [40]. On the contrary, technologies with an avatar and an agent such as VAPA allow for continued observation of the pulse rate.

In our study, patient adherence was moderate to high and higher than the 20–50% reported in previous studies [3,35], and patients continued their training in the follow-up period more frequently and with longer work-out sessions, suggesting a better adherence to continued training with an avatar–agent tele-rehabilitation set-up. However, it is important to note that the number of participants is decreasing as time passes. The ability of VAPA to align the exercise intensity to the pulse of the patient in real time and to guide the thresholds during each exercise set may explain the more positive behavioral changes seen with VAPA.

Our study has several limitations. The low number of participants and the drop-out rate may have influenced the results. However, this is not uncommon in patients with severe and advanced disease and not different from what is seen in rehabilitation programs for patients with other chronic respiratory diseases [41,42]. Our study was a pilot study to test the usefulness, and thus was delivered at a single center; we understand that practice may differ across regions and providers, hence a multicenter, prospective randomized study is needed to understand the place for online tele-rehabilitation in a range of clinical settings. Our study also has several strengths, such as the randomized design and the long-term follow-up. Further research is warranted to determine the optimal exercise training and identify strategies to maximize the long-term benefits in patients with IPF.

## 5. Conclusions

Tele-rehabilitation with VAPA appears to be useful for patients with IPF and the results are sustained exercise capacity at 3- and 6 months from baseline, while reduced exercise capacity was observed in the control group. No change in QoL or 7 days pedometry was observed. Tele-rehabilitation had a relatively high adherence as well as high patient satisfaction and safety. Tele-rehabilitation with VAPA thus seems to be a promising alternative rehabilitation strategy in IPF and a useful technology to increase IPF positive behavioral change towards a more physically active lifestyle.

## Figures and Tables

**Table 1:** The tele-rehabilitation program.

Features	Explanation
Video consultation sessions	Each patient met the physiotherapist in a video consultation to plan the rehabilitation program and to evaluate previous training experience.
E-learning packages	The patient had access to e-learning packages addressing psychological, medical, nutritional and physical aspects of IPF—in part supplied by relevant special data sources medicin.dk [19], lunge.dk [20] and helbredsprofilen.dk. [21] or created by dietitian students after in-depth interviews with pulmonary patients [22,23,24,25].
Chat sessions	Allowed the patient to interact and get prompt answers from the physiotherapist.
Workout sessions with VAPA	The patients trained 10–20 min 3–5 times a week at home with their individual and tailored VAPA using training aids such as elastics, weights and a fitness-step to reach the highest workout intensity. The VAPA provided encouragement to keep on training during the workout.
Questionnaires	The patients filled out questionnaires regarding satisfaction, breathlessness, and adverse events reporting.

**Table 2:** Baseline demographics of the 29 patients included in the study.

Variable	Tele-Rehabilitation with VAPA *n* = 15	Control *n* = 14
Male, *n* (%)	13 (86.6%)	8 (57.1%)
Age (years), mean (SD)	70.1 (8.8)	72.4 (7.6)
Months since diagnosis, median (IQR)	8.8 (0.0–20)	6.5 (0.0–7.5)
Smoking status Current, *n* (%) Former, *n* (%) Never, *n* (%)	3 (20%) 11 (73.3%) 1 (6.8%)	2 (14.3%) 9 (64.3%) 3 (21.5%)
Long-term oxygen therapy, *n* (%)	3 (20%)	0 (0%)
Antifibrotic treatment, *n* (%)	12 (80%)	14 (100%)
FVC (% predicted), mean (SD)	76.73 (16.4) *	90.8 (16.5) *
DLCO (% predicted), mean (SD)	46.46 (11.0)	55 (14.0)
6MWD (m), mean (SD)	461.5 (115.0)	446 (63.6)
7 days pedometry, mean (SD)	13,629 (5314)	11,883 (5237)
7dVMCPM, mean (SD)	480.3 (115.0)	412.5 (178.3)
SGRQ-I total, mean (SD)	49.8 (14.9)	47.7 (16.7)
KBILD total, mean (SD)	60.14 (12.1)	58.6 (10.0)
GAD7, mean (SD)	1.63 (2.5)	2.36 (3.9)

* *p* = 0.03; SD: Standard deviation; FVC: Forced vital capacity; DLCO: Diffusion capacity for carbon monoxide; 6MWTD: 6 min walk test distance; HR: Heart rate; 7 days pedometry: steps walked in 7 days; 7dVMCPM: 7 days vector magnitude counts per minute; SGRQ: Saint George Respiratory Questionnaire; KBILD: King’s Brief Interstitial Lung Disease Questionnaire; GAD7: General Anxiety Disorder-7 Questionnaire.

**Table 3:** Changes in primary and secondary endpoints within groups over time.

Variable	Baseline		Three Months		Six Months		NINE MONTHS	
	VAPA	Control	VAPA	Control	VAPA	Control	VAPA	Control
6MWTD	461.5 ± 115	446 ± 63.6	470 ± 115 *	421 ± 70 †	469 ± 136 *	423 ± 76 †	448 ± 133	390 ± 85
7 days pedometer	13,629 ± 5314	11,883 ± 5237	13,574 ± 8973	14,017 ± 9663	14,317 ± 12993	11,758 ± 6969	11,908 ± 7919	9936 ± 5804
7d VMCPM	480.3 ± 115	412.5 ± 178	444 ± 180	393 ± 186	408 ± 161	368 ± 182	426 ± 205	321 ± 151
SGRQ-I	49.8 ± 14.9	47.7 ± 16.7	51.2 ± 17.8	43.3 ± 16.4	48.3 ± 13.3	49.7 ± 22.2	43.9 ± 19.4	45.9 ± 16.6
K-BILD	60.14 ± 12.1	58.6 ± 10	60.5 ± 10	59.6 ± 13.0 ^†^	63 ± 11.8	54.1 ± 6.6	61.7 ± 10.8	59.5 ± 10.6
GAD7	1.63 ± 2.5	2.36 ± 3.9	3.27 ± 3.9	2.55 ± 3.3	2.9 ± 3.1	0.8 ± 1.7	2.1 ± 3.2	4.6 ± 3.7

Data are shown as mean ± SD. * Change from baseline significantly different from control group (*p* < 0.05); ^†^ *p* < 0.05 compared to baseline value within group; 6MWTD: 6-min walk test distance; 7dVMCPM: 7 days vector magnitude counts per minute; SGRQ-I: Saint George Respiratory Questionnaire; KBILD: The King’s Brief Interstitial Lung Disease Questionnaire; GAD7: General Anxiety Disorder-7 Questionnaire.

**Table 4:** Patient adherence shown as the number and percentage of training time performed and patient satisfaction from baseline to follow-up after 9 months.

		Expected	Trained	
**Patients**	**Period**	**Number**		**%**
15	0–3 Months	720 *	463 *	64
5	3–6 Months	720 *	775 *	108
3	6–9 Months	720 *	792 *	110

* Exercise minutes.

## Data Availability

Data is contained within the article or supplementary material.

## Supplementary Materials

The following are available online at www.mdpi.com/1660-4601/181/91/16/s1, File S1: Report 1 (Participants non participants; Report 2 (Baseline data); Report 3 (Follow up data).
