# Supplementary material for: Tele-Rehabilitation Program in Idiopathic Pulmonary Fibrosis—A Single-Center Randomized Trial
Source: Int J Environ Res Public Health. 2021 Sep 23;18(19):10016. doi: 10.3390/ijerph181910016 (PMC8508000; doi:10.3390/ijerph181910016)
Supplement: Supplementary file 1 [file ijerph-18-10016-s001.zip › ijerph-1367279-supplementary.pdf]

Supplementary

Report 1 (Participants non participants) **2**

Report 2 (Baseline data) **3**

Report 3 (Follow up data) 5

Report 1 (Participants non participants)

Table S1. Demographic data at baseline for participants and non-participants. Participants had longer 6MWT distance (+96.92 m) and higher absolute FVC (+0.68%).

	**NON-PARTICIPANTS**				**PARTICIPANTS**				**P-value**	**Mean Difference**
	**Mean**		**SD**	**n**	**Mean**		**SD**	**n**		
Age, years	75.29	±	6.37	17	71.41	±	8.26	29	0.11	3.88
6MWTD, m*	357.11	±	117.57	9	454.03	±	92.53	29	**0.03**	-96.92
FVC, l**	2.68	±	0.76	17	3.35	±	0.83	29	**0.00**	-0.68
FVC predicted%	84.59	±	16.27	17	83.52	±	17.65	29	0.84	1.07
DLCO***	7.71	±	1.39	16	8.32	±	1.29	25	0.19	-0.61
DLCO Predicted%	42.81	±	14.22	16	50.56	±	13.04	25	0.08	-7.75
Male/female	8 male / 9 female				21 male / 8 female					
* 6 Minute Walk Test Distance. ** Forced Vital Capacity *** Diffusing capacity for Carbon Monoxide										

Report 2 (Baseline data)

Table S1. Baseline demographics of all randomised patients.

		All patients (n = 29)
**Male, n (%)**		21 (72.41%)
**Age (years), mean (SD)**		70.88 (8.63)
**Months since diagnosis, median (IQR)**		7.3 (0.0-16.08)
**Smoking status**	***Current, n (%)***	5 (17.24%)
	Former, n (%)	20 (67%)
	Never, n (%)	4 (13%)
**Long-term oxygen therapy, n (%)**		3 (10.35%)
**Antifibrotic treatment, n (%)**		26 (89.65%)
**FVC (% predicted), mean (SD)**		83.52 (17.65)
**DLCO (% predicted), mean (SD)**		50.56 (13.04)
**6MWTD (m), mean (SD)**		454.03 (92.53)

SD:Standard deviation; IQR: Interquartile range; FVC: Forced vital capacity; DLCO: diffusion capacity for carbon monoxide; 6MWTD: 6 minutes walk test distance

Table S2. Baseline demographics in the control and intervention group.

		CONTROL			INTERVENTION			P-value*	Mean Difference
		Mean	SD	n	Mean	SD	n		
Pulmonary function	FVC%	90.79	± 16.45	14	76.73	± 16.42	15	**0.03**	14.05
	DLCO%	55.00	± 14.01	12	46.46	± 11.06	13	0.10	8.54
Physical performance	6MWTD, m	446.00	± 63.58	14	461.53	± 115.1	15	0.66	-15.53
	HR Restitution 2 min	18.71	± 8.57	14	19.50	± 13.50	14	0.86	-0.79
	Δ desaturation, % (0-6 min)	4.86	± 5.22	14	7.73	± 5.24	15	0.15	-2.88
	7dVMCPM	412.45	± 178.2	13	480.26	± 115.1	14	0.25	-67.81
	7 days pedometry	11883	± 5237	14	13629	± 5314	14	0.39	-1746.14
Quality of Life	SGRQ total	47.66	± 16.73	14	49.81	± 14.90	16	0.71	-2.14
	KBILD total	58.62	± 10.05	13	60.14	± 12.10	16	0.72	-1.52
	GAD7	2.36	± 3.92	14	1.63	± 2.53	16	0.54	0.73

* Independent t-test equal variances assumed

SD: Standard deviation; FVC: Forced vital capacity; DLCO: Diffusion capacity for carbon monoxide; 6MWTD: 6 minute walk test distance; HR: Heart rate; 7dVMCPM: 7 days vector magnitude counts per minute; SGRQ: Saint George Respiratory Questionnaire; KBILD: King's Brief Interstitial Lung Disease Questionnaire; GAD7: General Anxiety Disorder-7 Questionnaire.

Report 3 (Follow up data)

Follow up data

The control and intervention group were analyzed comparing the baseline data with data obtained after 3 months (end of rehabilitation), 6 months and 9 months (follow-ups).

Control group

Table S1: Changes over time in pulmonary function, physical performance, physical activity, exercise recovery and quality of life in the control group.

	**Baseline**			**3 months**			**6 months**			**9 months**			**p-value**<br>BL-3M<br>BL-6M<br>BL-9M
	**Mean**		**SD**	**Mean**		**SD**	**Mean**		**SD**	**Mean**		**SD**	
FVC, percent predicted	91.38	±	16.96	87.92	±	21.63	89.21	±	20.83	96.57	±	26.61	0.25<br>0.43<br>0.76
DLCO, percent predicted	57.90	±	13.46	51.90	±	14.31	55.33	±	15.84	50.83	±	10.48	**0.01**<br>0.42<br>**0.03**
6MWTD, m	456.00	±	51.73	420.80	±	70.16	422.62	±	76.48	389.57	±	84.80	**0.04**<br>**0.03**<br>0.21
7 days pedometry (steps)	13130.36	±	4991.65	14016.82	±	9663.48	11757.83	±	6968.53	9936.14	±	5804.07	0.79<br>0.69<br>0.56
7 d VMCPM	436.34	±	181.16	393.13	±	186.11	368.39	±	181.68	321.13	±	151.09	0.59<br>0.71<br>0.94
Desaturation	4.60	±	4.60	4.90	±	4.20	4.77	±	4.82	3.57	±	3.95	0.72<br>0.33<br>0.06
Difference between restitution 2min HR	18.00	±	9.89	17.10	±	10.82	20.58	±	11.74	15.57	±	23.71	0.82<br>0.39

													0.68
SGRQ total	47.92	±	16.96	43.27	±	16.43	49.71	±	22.17	45.89	±	16.61	0.54<br>0.61<br>0.60
KBILD total	57.43	±	11.01	59.59	±	13.02	54.10	±	6.57	59.46	±	10.57	0.30<br>**0.01**<br>0.46
GAD-7	2.73	±	4.36	2.55	±	3.33	0.83	±	1.70	4.60	±	3.75	0.92<br>0.26<br>0.98

SD: Standard deviation; FVC: Forced vital capacity; DLCO: Diffusion capacity for carbon monoxide; 6MWTD: 6 minute walk test distance; HR: Heart rate; 7dVMCPM: 7 days vector magnitude counts per minute; SGRQ: Saint George Respiratory Questionnaire; KBILD: King's Brief Interstitial Lung Disease Questionnaire; GAD7: General Anxiety Disorder-7 Questionnaire.

Intervention group

Table S2: Change over time in pulmonary function, physical performance, physical activity, exercise recovery and quality of life in the intervention group.

	**Baseline**			**3 months**			**6 months**			**9 months**			**p-value**<br>BL-3M<br>BL-6M<br>BL-9M
	**Mean**		**SD**	**Mean**		**SD**	**Mean**		**SD**	**Mean**		**SD**	
FVC, percent predicted	75.00	±	17.85	75.92	±	13.77	76.80	±	18.98	76.89	±	16.89	0.59<br>0.52<br>0.84
DLCO, percent predicted	48.14	±	14.16	46.86	±	12.85	46.14	±	13.46	44.00	±	11.97	0.34<br>0.91<br>0.58
6MWTD, m													0.57

	465.64	±	122.75	469.91	±	115.63	469.10	±	135.88	447.67	±	132.97	0.25<br>0.68
7 days pedometry (steps)	13860.00	±	5960.91	13574.09	±	8973.30	14316.75	±	12993.29	11907.89	±	7918.55	0.89<br>0.37<br>0.26
7 d VMCPM	487.36	±	125.37	444.24	±	180.34	408.31	±	160.72	425.84	±	205.34	0.35<br>0.77<br>0.78
Desaturation, %	8.55	±	5.87	8.18	±	5.13	8.90	±	5.59	7.67	±	4.33	0.51<br>0.21<br>0.37
Difference between restitution 2min HR	20.00	±	15.42	19.38	±	11.38	19.38	±	12.20	18.50	±	11.64	0.92<br>0.84<br>0.96
SGRQ total	47.62	±	9.86	51.16	±	17.83	48.26	±	13.31	43.91	±	19.42	0.62<br>0.87<br>0.74
KBILD total	63.34	±	12.72	60.52	±	10.03	63.01	±	11.77	61.68	±	10.77	0.36<br>0.65<br>0.86
GAD-7	1.82	±	2.96	3.27	±	3.88	2.88	±	3.14	2.10	±	3.25	0.21<br>0.58<br>0.95

SD: Standard deviation; FVC: Forced vital capacity; DLCO: Diffusion capacity for carbon monoxide; 6MWTD: 6 minute walk test distance; HR: Heart rate; 7dVMCPM: 7 days vector magnitude counts per minute; SGRQ: Saint George Respiratory Questionnaire; KBILD: King's Brief Interstitial Lung Disease Questionnaire; GAD7: General Anxiety Disorder-7 Questionnaire.

Differences between groups

Table S3. Differences between the control and intervention groups in pulmonary function, physical performance, physical activity, exercise recovery and quality of life after treatment and follow-ups.

	**Mean Difference**	**Std Error Difference**	**Mean Difference**	**Std Error Difference**	**Mean Difference**	**Std Error Difference**	**p-value**<br>3M<br>6M<br>9M
	**3M**		**6M**		**9M**		
FVC, percent predicted	-4.38	3.39	-3.27	3.14	-1.98	5.01	0.21<br>0.31<br>0.70
DLCO percent predicted	-4.71	3.63	-2.92	3.87	-3.17	2.42	0.12<br>0.46<br>0.22
6MWTD, m	-39.47	16.18	-34.28	13.33	-40.54	26.83	**0.03**<br>**0.02**<br>0.15
7 days pedometry (steps)	1172.36	3725.48	-1756.17	3643.27	-998.32	3384.67	0.76<br>0.64<br>0.68
7 d VMCPM	-0.08	88.95	4.72	90.33	-64.13	75.85	1.00<br>0.96<br>0.41
Desaturation	0.66	0.94	-0.91	1.02	0.08	1.40	0.49<br>0.38<br>0.41
Difference between restitution 2min HR	-0.28	6.74	1.50	6.05	-3.80	10.52	0.97<br>0.69<br>0.72
SGRQ total	-8.18	10.10	2.76	10.16	0.00	7.63	0.43<br>0.79<br>1.00
KBILD total	4.98	3.61	-0.61	2.62	3.22	4.44	0.18<br>0.82<br>0.48

							0.43
GAD-7	-1.64	2.04	-2.42	2.00	2.70	2.28	0.45
							0.25

SD: Standard deviation; FVC: Forced vital capacity; DLCO: Diffusion capacity for carbon monoxide; 6MWTD: 6 minute walk test distance; HR: Heart rate; 7dVMCPM: 7 days vector magnitude counts per minute; SGRQ: Saint George Respiratory Questionnaire; KBILD: The King's Brief Interstitial Lung Disease Questionnaire; GAD7: General Anxiety Disorder-7 Questionnaire.

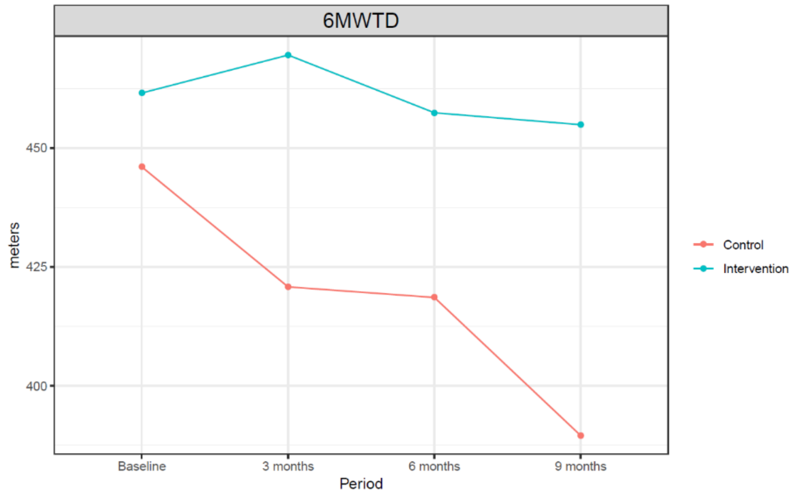

Figure S1. Mean six minute walk test distance in the control and intervention group (meters).

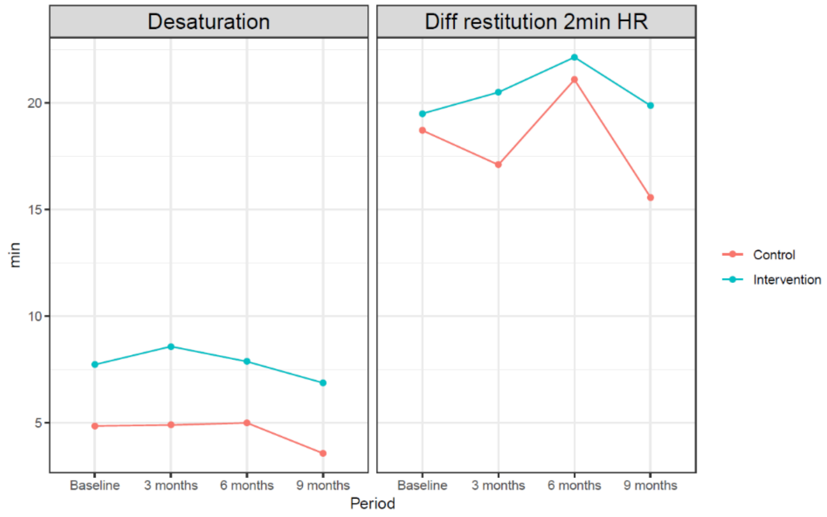

Figure S2. Mean exercise recovery time for the control and intervention group (minutes).

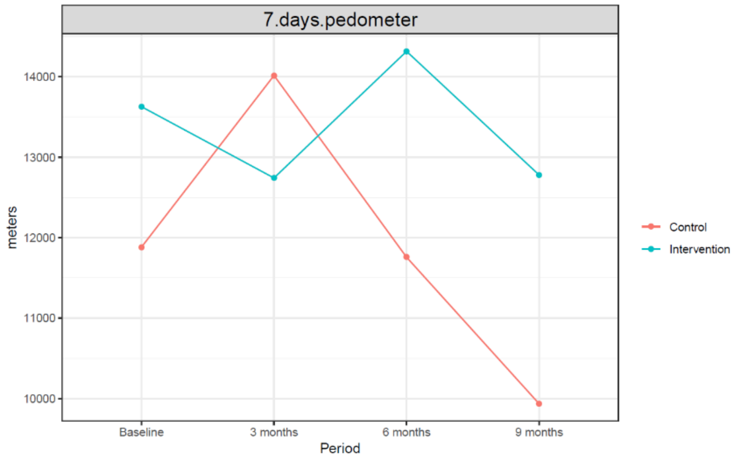

Figure S3. Mean 7 days pedometry over time for the control and intervention group (meters).

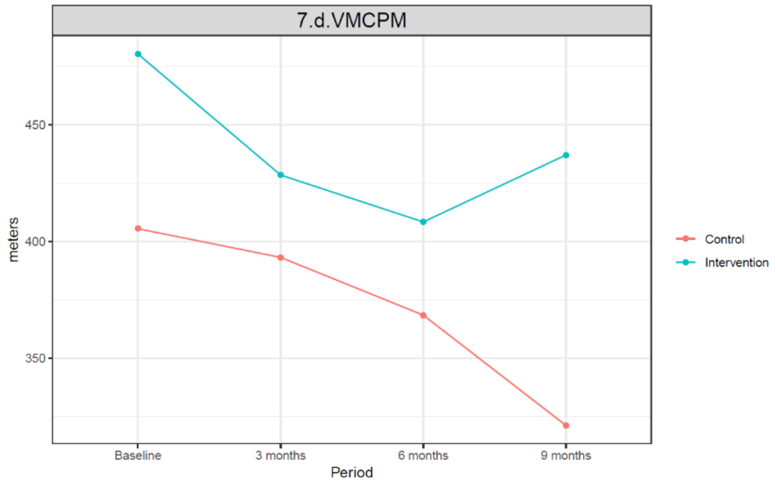

Figure S4. Mean 7 dVMCPM over time for the control and intervention group (meters).

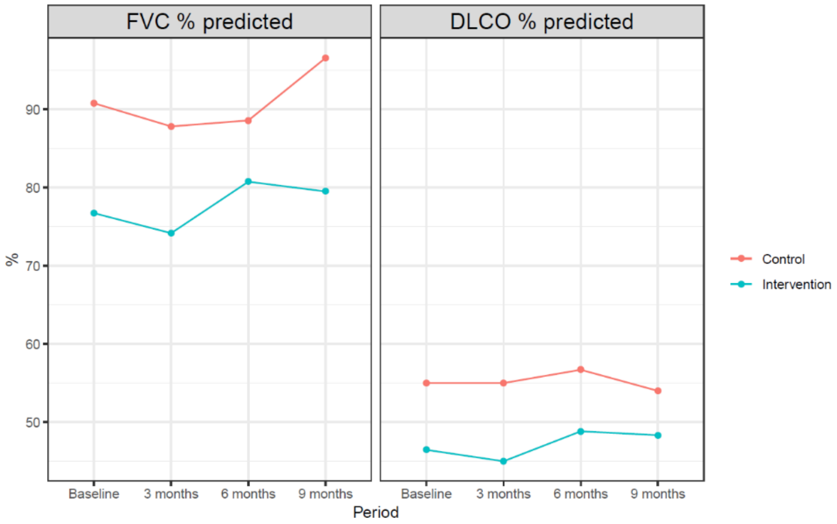

Figure S5. Mean FVC percent predicted and DLCO percent predicted over time for the control and intervention group.

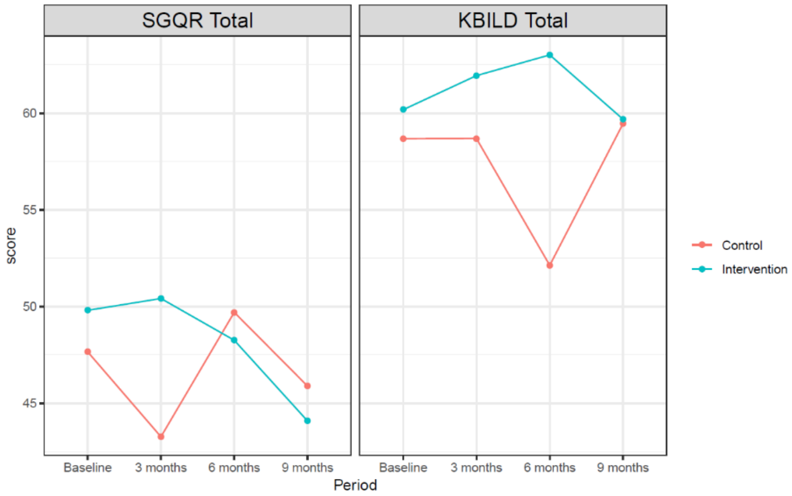

Figure S6. Mean SGRQ and KBILD total score over time for the control and intervention group.

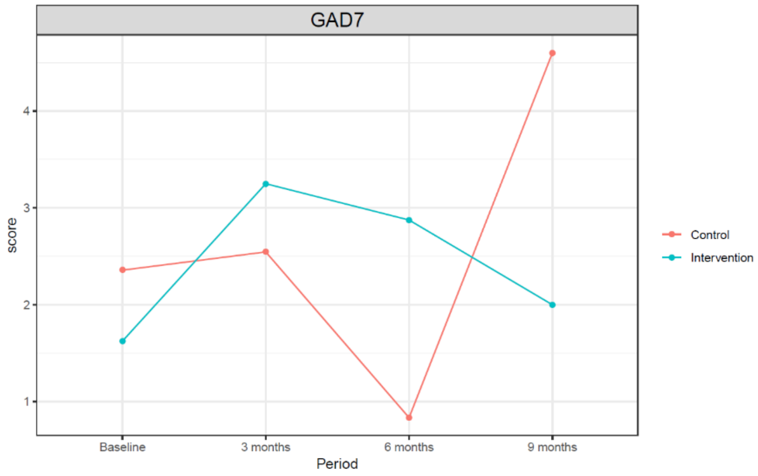

Figure S7. Mean GAD-7 over time for the control and intervention group.
